# Improving Health by Investing in Medical Education

**DOI:** 10.1371/journal.pmed.0020424

**Published:** 2005-12-27

**Authors:** The PLoS Medicine Editors

## Abstract

Medical education in South Asia is reinventing itself, to create doctors who can meet the needs of the local community.

One of the common criticisms of medical education is that there is often a mismatch between what is taught at medical school and the actual skills that are needed by doctors to provide locally relevant health care. This mismatch is particularly striking in South Asia, according to the 60 educators who gathered for the Second International Consultation on Undergraduate Medical and Pharmacy Education, held in Negombo, Sri Lanka, in September 2005. The aim of the consultation, organized by the health-activist group Health Action International Asia Pacific and the World Health Organization, was to consider how best to prepare medical students to meet the health needs of the region.

The needs seem overwhelming. South Asia faces high rates of communicable and noncommunicable diseases, road traffic injuries, maternal and child mortality and morbidity, rising tobacco use, violent conflicts, and the devastating effects of recent floods and earthquakes. And yet there is hope. Zulfiqar Bhutta and colleagues have argued that “the answers to the region's problems may already be with us” (BMJ 328: 777–778). They point out that despite a civil war, Sri Lanka has the best health indicators in the region—better than those of most other countries with comparable incomes. Sri Lanka's average life expectancy is 73 years, infant mortality is 16 per 1,000, and maternal mortality is 30 per 100,000 live births. India's Kerala state has similarly impressive health indicators, which are better than the national average. These two examples show what can be achieved when governments spend their limited resources on education (leading to high literacy rates) and on providing community-based primary care, rather than building expensive specialist hospitals.

If all countries in the region are to emulate the success of Sri Lanka and Kerala, they will need, among other things, to reorient their medical schools away from teaching students in acute hospital settings and toward community-based education. At the Negombo consultation, Qasem Chowdhury, Vice Chancellor of the Peoples' University-Gono Bishwabiddyaloy, Institute of Health Sciences, Bangladesh, laid out the challenge. Meeting the health needs of South Asia, he said, “requires a new type of educational program for health personnel that will make them responsive to the needs of the majority population of individual countries. Such training is most effective if it is carried out in close relation to the actual community in which health personnel are later to work.”

The educators at the consultation were united in calling for curricular reform. The traditional curriculum—overloaded with basic sciences teaching, focused on curative rather than preventive medicine, emphasizing factual learning rather than acquisition of skills, knowledge, and attitudes—is inappropriate for South Asia. And the neglect of social, economic, cultural, and political perspectives in undergraduate training means that doctors can never conduct their practice with an understanding of the fundamentally social nature of disease in the region.

Several inspiring examples of curricular reform were presented, such as that of the University of Sri Jayewardenepura, Sri Lanka. With support from the World Bank and Sri Lanka's Ministry of Higher Education, the university is adopting a new medical curriculum that integrates basic and clinical sciences, and emphasizes community-based learning and capacity building for research. The university is establishing “laboratories” for learning clinical skills, communicating with patients, and using information technology for self-directed learning and clinical practice. Clearly, different medical schools in South Asia will have different resources at their disposal for overhauling their curricula (the Afghani delegates at the meeting, for example, were desperately short of textbooks), but, nevertheless, all can take at least tentative steps toward problem-based, community-oriented, integrated teaching.

What other steps should schools take to produce doctors who can appropriately serve their communities? The same answers recurred throughout the consultation. Schools should assess students not just on knowledge but on broader skills that are essential for promoting community health. Students should be taught about rational drug prescribing, medical ethics and human rights, and the traditional systems of medicine that are hugely popular among their patients. And faculty development (training the teachers in educational skills) must be at the heart of the reforms.

When a consultation ends, the real work begins. Although there was a consensus about what kind of change is needed, there are many unanswered questions about the best way to bring about these reforms. One contentious question, for example, is whether the private sector has a role to play in providing medical education, particularly in parts of South Asia where public education is failing. How can such a question be answered? One suggestion at the consultation was to establish a regional network of community-based educators, to allow educators to share their experiences of—and research on—their educational reforms. We look forward to providing an update on these concerns in a future issue of *PLoS Medicine*.

